# Microfluidic and Nanofluidic Resistive Pulse Sensing: A Review

**DOI:** 10.3390/mi8070204

**Published:** 2017-06-25

**Authors:** Yongxin Song, Junyan Zhang, Dongqing Li

**Affiliations:** 1 Department of Marine Engineering, Dalian Maritime University, Dalian 116026, China; yongxin@dlmu.edu.cn (Y.S.); junyan@dlmu.edu.cn (J.Z.); 2 Department of Mechanical and Mechatronics Engineering, University of Waterloo, Waterloo, ON N2L 3G1, Canada

**Keywords:** resistive pulse sensing, particle sizing and counting, microfluidics and nanofluidics, review

## Abstract

The resistive pulse sensing (RPS) method based on the Coulter principle is a powerful method for particle counting and sizing in electrolyte solutions. With the advancement of micro- and nano-fabrication technologies, microfluidic and nanofluidic resistive pulse sensing technologies and devices have been developed. Due to the unique advantages of microfluidics and nanofluidics, RPS sensors are enabled with more functions with greatly improved sensitivity and throughput and thus have wide applications in fields of biomedical research, clinical diagnosis, and so on. Firstly, this paper reviews some basic theories of particle sizing and counting. Emphasis is then given to the latest development of microfuidic and nanofluidic RPS technologies within the last 6 years, ranging from some new phenomena, methods of improving the sensitivity and throughput, and their applications, to some popular nanopore or nanochannel fabrication techniques. The future research directions and challenges on microfluidic and nanofluidic RPS are also outlined.

## 1. Introduction

Accurately determining the size and number of particles and cells in electrolyte solutions is an important task in many fields, such as biomedical research [1,2,3,4,5,6], clinical diagnosis [7,8,9,10,11,12], and environmental monitoring. Among the methods for particle counting and sizing [13], resistive pulse sensing (also called the Coulter principle) probably is the most popular method. This method was invented by Coulter [14], aiming to replace manual cell counting with an automatic device. In a Coulter counter, a small insulating orifice is immersed into an electrolyte solution with suspended particles. A direct current (DC) voltage is applied through two electrodes placed across the orifice and an electrical current is conducted by the electrolyte solution and the orifice creates a ‘‘sensing zone’’. When a particle passes through the orifice, due to the different resistivity of the particle and electrolyte solution, a temporary electrical resistance change across the orifice is generated. This change is measured in terms of a voltage or a current pulse, whose magnitude is proportional to the volume of the particle for a given orifice. With the invention of Coulter counter, flow cytometry, which is widely used for detecting, counting, sizing cells and particles with a throughput of thousands of cells per second [15,16,17,18,19,20,21,22], became available [23]. The traditional flow cytometer, however, is bulky in size due to bulky pumps, tubes, valves, and other auxiliary components. Furthermore, it is expensive and cannot handle small volumes of samples.

With the advancement of Lab-on-a-chip technologies [24,25,26,27,28,29,30], microfluidic and nanofluidic RPS sensors with high sensitivity and accuracy were developed. Besides the basic functions for particle sizing and counting, a nano-RPS sensor can also characterize nanoparticles, DNA, viruses, antigens and so on. Such advancements greatly enrich the powerful abilities of the RPS technology and make the development of a low cost and portable flow cytometer possible.

This paper aims to review the latest development of microfuidic and nanofluidic RPS technologies within the last 6 years, ranging from some new phenomena, nanochannel or nanopore fabrication technologies, methods of improving the sensitivity and throughput, as well as their applications. The paper begins by introducing some basic theories of particle sizing and counting of microfluidic RPS sensors. Then, some interesting phenomena and applications of microfluidic and nanofluidic RPS are reviewed. At the end, the future research directions and challenges on microfluidic and nanofluidic RPS are discussed. 

## 2. Working Principle and New Sensing Phenomena

**Figure 1:**
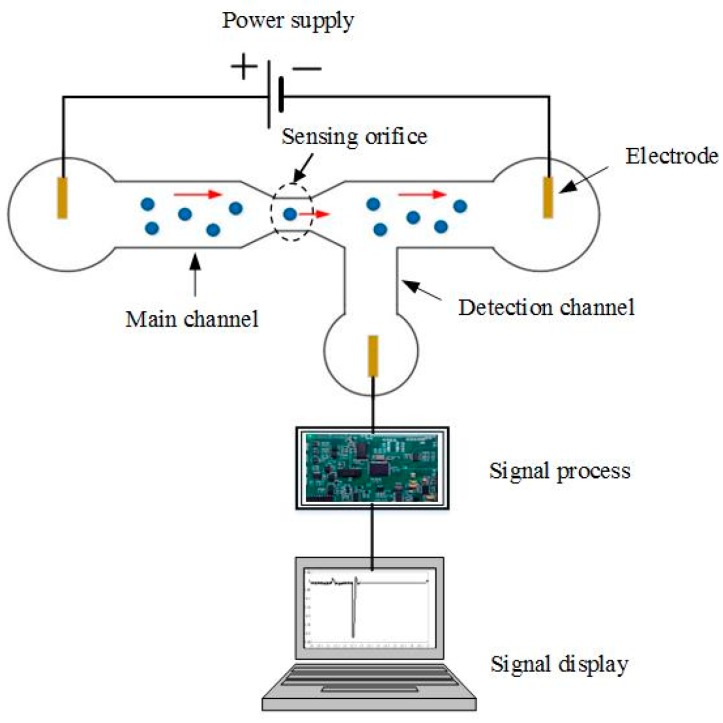
Working principle of a microfluidic resistive pulse sensing (RPS) system.

Microfluidic and nanofluidic RPS employ the principle of the Coulter counter in microfluidic or nanofluidic channels for particle counting and sizing. The working principle and typical system setup is shown in Figure 1. For the system shown in Figure 1, an electrical field is applied across a sensing orifice whose size is much smaller than the main channel which is filled with an electrolyte solution. Each particle passing the sensing orifice will generate a resistive pulse which is processed by the amplification circuit, the data acquisition device, and the computer. Each particle will generate one signal pulse and the magnitude of the signal represents the volume ratio of the particle and the sensing gate. In this way, particle sizing and counting are achieved.

It should be noted that both DC and alternative current (AC) fields can be applied (named as DC RPS and AC RPS, respectively). For the DC RPS, the system is very simple without using the bulky AC power source and the complicated signal processing circuit. However, the polarization of the electrode in electrolyte solution will increase the electric resistance of the main channel and thus is adverse to the sensitivity of the system. [31,32,33]. Such polarization effects can be avoided by using an AC electric field which can decrease the polarization resistance by increasing the excitation frequency [33]. In order to minimize the stray capacitance effects [34], however, appropriate frequency must be carefully determined depending on the specific chip design [31,32,35,36,37].

For particle sizing with the RPS method, the most important question is how to evaluate particle’s size based on the detected signal. That is, the relationship between the measured magnitudes of signals and the sizes of the sensing orifice (or sensing channel) and particles must be determined. Such a relationship can be determined by calculating the resistance change caused by a particle entering the sensing channel (Δ*R*). Table 1 summarizes the relative electrical resistance change caused by spherical particles with different diameters.

It should be noted that the equations shown in Table 1 were derived based on the assumption that a spherical particle moves along the centerline of a cylindrical microchannel. With the advancement of micro and nanofabrication technology, different types of micro and nanochannels were available and new RPS phenomena in sub-microscale and nanoscale have been demonstrated recently. One interesting phenomenon recently discovered is the generation of a double-peak (resistive-and-conductive peak) when a particle passes through the sensing pore [44,45]. Such a phenomenon is totally different from the classical Coulter theory. Menestrina et al. [45] used a polyethylene terephthalate (PET) pore (12 μm long and 500 nm–1.5 μm in diameter) for RPS detection of particles of several hundred nanometers in KCl solution with different concentrations. They found that double peaks, first a downward resistive peak and then an upward conductive peak, will be generated when the concentration of the KCl solution was below 300 mM. Through numerical simulations and experimental verifications, the authors concluded that the positive peak (conductive) is caused by the modulated ionic concentrations induced by the charged particles entering into the sensing pore. 

Weatherall et al. [44] also experimentally investigated the double-peak phenomenon when measuring 200 nm carboxylate polystyrene spherical particles with a tunable RPS method. They found that the onset electrolyte concentration for double-peak formation is about 50 mM. More importantly, the formation of a double-peak is due to the ionic concentration polarization. They also found that the shape of the double-peak depends both on the moving directions of the particle and the applied voltages. For example, a positive voltage bias and a positive hydraulic pressure will only generate a resistive pulse. With a negative voltage bias and a positive pressure, however, a conductive and resistive pulse will be generated. 

Considering the ‘end effects’ of the sensing pore, Willmott et al. [46,47] built up a semi-analytic model to simulate the shapes of the pulses. They found that end effects are prominent when the center of the particle is less than one radius inside the pore entrance. They also found that asymmetry is more obvious for larger and slower moving particles. Such results are valuable for evaluating a particle’s size based on the measured pulse because most of the sensing pores are not of the ideal shape with a constant cross-section in practice.

The geometry effects of a nanopore on RPS nanoparticle sensing were also investigated. It was found that the length [48], thickness [49], tip diameter [50], and base ratio [51] of the silicon nitride membrane nanopores can greatly influence the electric field around the ends of the nanopores. More recently, Kaya et al. studied the influence of the cone angle of a conically shaped poly(ethylene terephthalate) (PET) membrane nanopore on DNA sensing. They found that the electric field at the tip of the nanopore increases with the increase in the cone angel. As a result, the magnitude of the pulse will also be increased with the increased cone angel [52].

Beside the geometry of the nanopores, the surface charges at the interface of nanopore–electrolyte solution can also influence the magnitude and duration of a RPS signal pulse. Liu et al. [53] showed that the surface charges can either increase or decrease the magnitude of the signal pulse depending on the positive or negative potential bias applied across the sensing channel. Recently, Weatherall et al. [54] found that particles near the edge of a tunable nanopore entrance generate larger RPS signals. The smallest RPS signal is generated by a particle moving along the center line of the nanopore.

Recent studies also showed that both the shape and material of a particle can influence the detected RPS signals. For example, using a rod-like Au particle (several micrometers in length and several hundred nanometers in diameter) and a tunable elastomeric membrane pore, Platt et al. [55] studied the effects of shape of the particle on RPS signals. They found that the full width at half maximum of the RPS signal can be used to characterize the rod length. More importantly, they showed that for the rod-like particles, the measured current change remains unchanged or slightly increased with the increase in the pore size. However, the current change caused by a spherical particle is decreased. 

**Figure 2:**
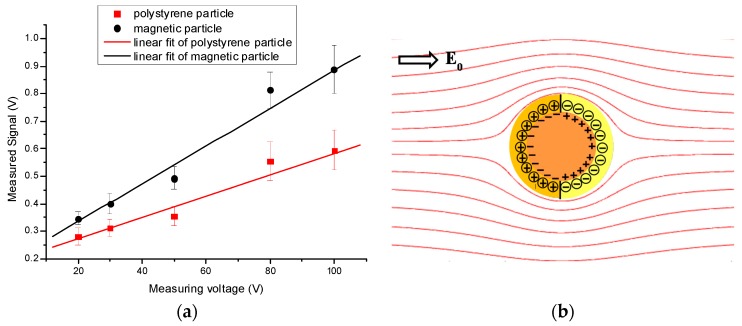
(**a**) Dependence of the magnitudes of measured RPS signals on the applied voltages and (**b**) the induced electrical double layer.

Lee’s study showed that the magnitude of a RPS signal generated by an elongated nanoparticle is larger than that by a spherical nanoparticle of the same volume [56]. Song et al. [57] studied the effect of induced surface charge of metal particles on RPS particle sizing. The experimental results showed that the magnitude of the RPS signals generated by 5 μm magnetic particles is larger than that of a 5 μm polystyrene particle under the same condition, as is shown in Figure 2. Such results are due to the thicker electrical double layer (EDL) formed by the induced charges around the magnetic particles. 

These new phenomena mentioned above are important and not covered in the traditional RPS theory. Such phenomena should be well considered for particle size evaluation with the RPS method. 

## 3. Methods for Throughput and Sensitivity Improvement 

### 3.1. Throughput

One key advantage of microfluidic RPS is its simplicity, because the bulky pumps and valves are no longer needed due to the using of a micro- or nanoscale-sized channel or sensing pore and thus a greatly decreased sample volume. Therefore, microfluidic and nanofluidic RPS is very promising to be developed into portable lab-on-a-chip (LOC) devices. However, the throughput of a portable device is relative low due to the slow sample transportation velocity in a microfluidic channel. The sensitivity is also limited by the limited ability of fabricating a small sensing channel or pore and the simple data processing circuit. For microfluidic and nanofluidic RPS, the throughput and sensitivity are generally coupled with each other. A smaller sensing channel or sensing pore is needed in order to have a higher sensitivity. Achieving this, however, will lower the throughput due to the constraint of using the smaller sensing channel.

To improve the counting throughput, Jagtiani et al. [32] developed a frequency division multiplexing method which can encodes each channel with a specific frequency with a single pair of electrodes and process the signals with a single electronic system. Such a method enables monitoring multi-channels individually and obtaining an 300% improvement of throughput over a single-channel device. For this design, however, each channel needs one specific signal process channel to decode the signals.

Following a similar idea, Liu et al. [58] designed novel coplanar electrodes that can generate orthogonal digital codes when particles pass over the electrodes. The orthogonal digital codes were then decoded by the Code Division Multiple Access (CDMA) principle. Such a design, which can even differentiate the overlapped signals with >90% accuracy, can be easily extended to orthogonally counting particles in an arbitrary number of microfluidic channels and thus provides a very promising approach for solving the throughput problem of a microfluidic flow cytometer.

**Figure 3:**
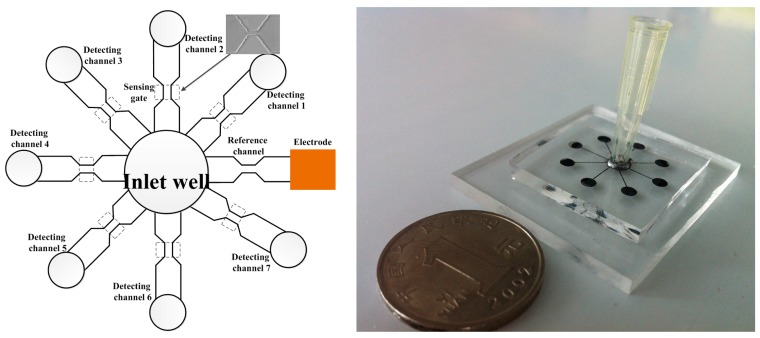
The structure of the high throughput microfluidic chip.

Song et al. [59] also put forward a novel method to improve the throughput of a DC RPS device. They designed a sensitive differential microfluidic sensor with multiple detecting channels and one common reference channel (Figure 3). With seven detecting channels, an average throughput of 7140 cells/min under a flow rate of 10 μL/min was achieved. Counting throughput can be further increased by increasing the number of the detecting channels. It should be noted that a reference channel makes a good balance between the sensitivity and the throughput.

Rather than putting efforts on chip design innovation, Castillo-Fernandez et al. [60] adopted a signal post-treating approach to improve the throughput. They designed an electronic system that allowed a counting throughput of about of 500 counts/s.

### 3.2. Sensitivity

Theoretically, the sensitivity of the RPS sensor is mainly determined by the volume ratio of the particle and the sensing channel as well as the noise level of the electronic system. Therefore, sensitivity can be improved either by increasing the volume ratio or by using advanced signal processing instruments to cancel the noise. 

One way to increase the volume ratio is to decrease the volume of the sensing channel. For smaller particle detection, such as DNA, proteins, and viruses, a nano-sized sensing channel or pore is needed. The details about the methods of fabricating such channels or pores will be reviewed and discussed in Section 5. Here, several other novel methods on decreasing the volume of the sensing channel are reviewed first.

One novel and simple way is to use less conductive focusing solutions to decrease the volume of the sensing channel. Choi et al. [61] used hexadecane as the focusing solution to hydraulically control the width of the passage for the sample solution. The sample solution is focused by the focusing solution (hexadecane) to pass the sensing region. Due to the very low conductivity of hexadecane, the electric field only exists in the sample solution and becomes concentrated in the focused region. In this way, the volume in the sensing region is narrowed and can be flexibly controlled by the flow rate of the focusing solution. Such a RPS sensor has a controllable range of sensitivity and was successfully applied for submicron-sized bacterial detection with a 30 μm wide detection channel.

One problem associated with using oil as the focusing solution is that the large surface tension at the oil–water interface can make the flow unstable. As a result, the sensing volume will also be changing and thus noise level is increased too [62]. To solve this problem, Bernabini et al. added some surfactants into the oil (focusing solution) to stabilize the oil–sample solution interface. Due to the increased stability of the sensing volume by using an oil/surfactant mixture, detection of 1 μm polystyrene particles and discrimination of bacteria and polystyrene particles of similar size with a 200 μm wide sensing channel were achieved [62].

**Figure 4:**
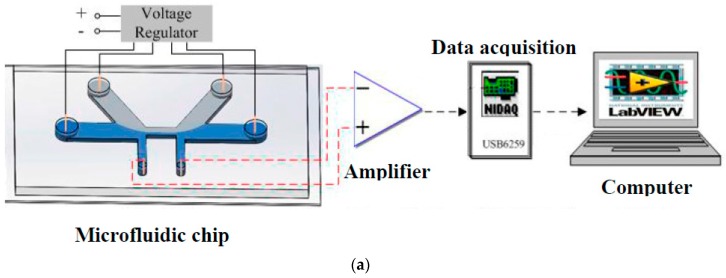

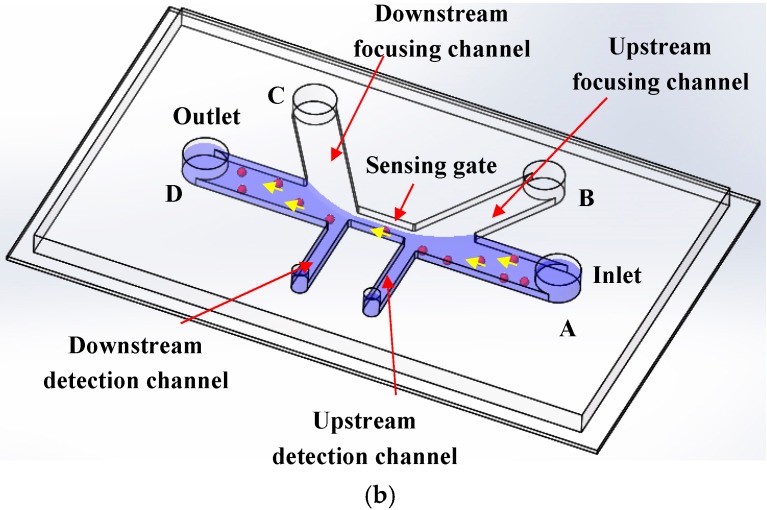
(**a**) A schematic diagram of the system setup, and (**b**) the structure of the microfluidic chip.

**Figure 5:**
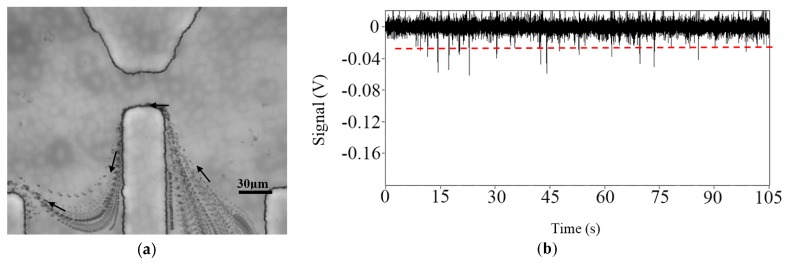
(**a**) Typical flow trajectory, and (**b**) detected RPS signals of 1 μm particles (V_A_ = 27 V, V_B_ = 52 V, V_C_ = 0 V and V_D_ = 24 V).

For the above two studies, it should be noted that they used two or four electrodes to apply an AC electric field and the sample solutions were transported by pressure-driven flow. For these designs, the distance between the electrodes defines the length of the sensing region. The focusing solution was only used to decrease the width of the sensing region. More recently, Liu et al. [63] reported a novel electrokinetic-flow-focusing method to narrow the size of the sensing channel and thus improve the sensitivity. This method is particularly useful for a DC RPS system with an elelctrokinetically-driven flow. Figure 4 shows the structure of this novel microfluidic RPS sensor and its working principle. The microfluidic chip has inlet and outlet wells, one main channel, two detecting channels, two focusing channels, and the corresponding wells. The key feature of this sensor is that the focusing solution can flow only from the upstream focusing channel to the downstream focusing channel. In this way, the sensitivity is greatly improved. As a result, detection of 1 μm with a physical sensing gate of 30 μm × 40 μm × 10 μm (width × length × height) was successfully achieved (Figure 5). 

## 4. Applications

With the improved RPS sensitivity, especially the ability of fabricating a nano-RPS sensor, label-free particle detection in nanoscale, such as detection of DNA, proteins, viruses, and so on, were possible. With the measured RPS signals, nanoparticle characterization was also reported. In this section, we will review the recent developments on the typical applications of RPS technology in submicro- and nanoscale.

### 4.1. DNA Detection and Analysis

With the first demonstration of RPS detection of single stranded DNA detection by a biological α-hemolysin pore [64], nano-RPS now is an attractive tool for label-free DNA detection with the distinctive advantages of its simplicity and high sensitivity [65,66,67,68,69,70,71]. The commercially available qNano device exemplifies the strength of this technology.

Among the recent investigations, the mostly common used approach of DNA detection with RPS is to detect the change caused by the binding of DNA to a particle. For example, the change of the width and frequency of the RPS signals caused by the binding of thrombin to DNA-coated magnetic beads [67], the change in the full-width half maximum (FWHM) caused by DNA strands hybridized with the probe-grafted magnetic particles [72], have been used for DNA identification.

To increase the small size difference by the adsorption of DNA, an approach of amplification was adopted by Kühnemund [73] and Yang [70]. The key idea is to increase the amount of DNA and thus increase the signal difference and detection sensitivity. Firstly, target DNA, together with padlock probes and capture oligo, is captured by the magnetic particle. After amplification, the magnetic particles are measured with the RPS system. For the particles with target DNA, it will generate signals with larger magnitude and wider FWHM. 

In 2013, Traversi et al. [74] fabricated graphene and SiNx nanopores with which the electrokinetic translocation behavior of pNEB DNA and λ-DNA was measured. Since the graphene membrane can be as thin as 0.0335 nm, comparative to the distance between two bases in a DNA chain, it is promising for fabricating a DNA sequencing device. Hernándezainsa et al. [75] fabricated a glass nanocapillary (with a central needle hole) and studied the structures of DNA origami using both visual observation and RPS detection. This demonstrates the possibility of selective detection of double-strand DNA and thus the great potential for DNA sequencing with the RPS method. More recently, Sischka et al. [76] studied the translocation force on DNA molecules in nanopores. It was found that the membrane thickness had a great influence on the translocation of DNA molecules.

### 4.2. Label-Free Protein Detection

With the improved sensitivity for particle sizing, especially the ability to fabricate a nano-RPS sensor, label-free detection of a specific kind of nanoparticles, such as proteins and DNA, from a mixture solution becomes possible. Rodriguez-Trujilloa et al. [77] measured the specific protein concentrations in a suspension with a microfluidic RPS system. Briefly, bead oligomers were formed when the two functionalized polystyrene beads and the rat IgG serum were mixed together. Such oligomers are larger in size and will generate RPS signals with larger magnitude. By comparing the magnitudes of the detected signals, the presence of the target protein can be easily determined. However, more single bead will be available with the increased protein concentration. The authors attributed such results to the binding and blocking of more sites on the surface of the two beads under high protein concentration.

Following a similar idea, RPS detection of a cancer biomarker [78], human ferritin [79], and even single-molecule proteins [80] were also demonstrated. It should be emphasized that for RPS detection, any specific binding will be reflected by the detected electric signals. Thus, fluorescent labeling is no longer needed. This is much more advantageous and attractive in saving time and simplifying detection.

### 4.3. Nanoparticle Characterization

The nano-RPS device is a new, yet powerful tool for nanoparticle analysis. Based on a single-particle measurement, much useful information, such as size and concentration distribution, surface charges, and even translocation behavior can be reliably obtained [56,81,82,83,84,85,86].

Vogel et al. [81] demonstrated the accuracy and reliability of sizing nano-sized particles with a tunable RPS sensor made of polyurethane. The nano-RPS system was fabricated by mounting a resizable elastomeric thermoplastic polyurethane (TPU) membrane on the q-Nano device. The membrane was stretched open to different pore sizes. The experimental results showed that the sizes of different particles, for example polymethyl methacrylate (PMMA) and nonfunctionalized polystyrene particles, adenovirus, and so on, can be accurately evaluated based on the pre-obtained calibration curve. Following a similar calibration methodology, Roberts et al. [82] measured the concentrations of the marine photosynthetic cyanobacterium Prochlorococcus (with a diameter of 600 nm) by using a tunable polyurethane nanopore. A good agreement with the results from microscopy counting was found. Pal et al. [85] measured the size distribution and concentration of engineered nanomaterials in cell culture media with a tunable nanopore RPS method. They found that TRPS technology offers higher resolution and sensitivity compared to the dynamic light scattering (DLS) method. Due to the high accuracy and resolution in sizing nanoparticle, the nanopore RPS sensing technique was applied to characterize the swelling of pH-responsive, polymeric expansile nanoparticles by Colby et al. [83], and to measure the size and the surface charge of silica nanoparticles in serum by Sikora et al. [87]. Compared with the results from other methods, such as transmission electron microscopy, differential centrifugal sedimentation (DCS), and dynamic light scattering (DLS), the tunable RPS sensor showed excellent performance in sensitivity and resolution.

Fraikin et al. [84] developed a polydimethylsiloxane (PDMS) high-throughput nanoparticle RPS sensor that can size and determine the concentration of a nanoparticle suspension and unlabeled bacteriophage T7 in both salt solution and mouse blood plasma with a throughput of about 500,000 particles per second. With this sensor, the authors also firstly discovered some naturally occurring nanoparticles in the native blood plasma. Through both finite-element simulations and experimental verification, Lee investigated the geometry of the pore and the particle on the magnitudes of the detected RPS signals. The results showed that a conical pore can generate larger signals in magnitudes than a cylindrical pore. More importantly, both the size and shape of the nanoparticle can be simultaneously determined based on the magnitude and the *y*-position of 10% resistive pulse (*y* 10%) [56].

Besides sizing the bare nanoparticle, estimation of the thickness of the protein layer absorbed onto the nanoparticles in serum was also achieved, using a tunable RPS method by Sikora et al. [87]. Recently, Luo et al. reviewed the advances in the transport motion of a single nanoparticle by the RPS method, with an emphasis on the forces governing the translocation of low-aspect-ratio, non-deformable particles [86].

Determining the zeta potential, an important electrokinetic parameter, of carboxylated polystyrene nanoparticles can also be reliably achieved with the RPS method [88,89,90,91,92]. Eldridge et al. [89] measured the zeta potential of carboxylated polystyrene nanoparticles. The idea is to find the critical pressure at which the drag forces of the pressure-driven flow and electroosmotic flow and the electrophoretic force exerted on the nanoparticle become balanced. Once balanced, the frequency of the signal pulse becomes minimum which can be used to calculate the electrophoretic mobility of the particle (also the zeta potential). By using the same system and the same measuring principle, Somerville et al. [90] measured the zeta potential of a water-in-oil emulsion, demonstrating the ability of measuring zeta potential of a single soft particle. To further evaluate the accuracy of this technique, Eldridge et al. [89] conducted experiments to measure particles with different diameters and surface charges and concluded that the full width half maximum (FWHM) duration of a signal pulse is more reliable in determining zeta potential. More recently, determining the zeta potential of DNA modified particles, discrimination of ssDNA, dsDNA, and small changes in base length for nucleotides were reported by Blundell et al. [69]. All of the above investigations demonstrate the power of the RPS technology to characterize bio-nanoparticles with high sensitivity and resolution. 

## 5. Fabrication of the Nano-Sensing Gate 

As reviewed above, there is an increasing interest in nano-RPS technology nowadays, propelled both from the interests in scientific understanding and promising applications in nanoscale. For a nano-RPS device, the most important component is the sensing orifice, which is normally in form of a nanopore or a nanochannel. Therefore, advancement in nanofabrication is vital for the development of a nano-RPS device. In addition to the several well-known review papers [86,93,94,95], we will review the latest development of fabricating two typical nanosensing orifices: nanopores and PDMS nanochannels. 

### 5.1. Nanopores

The nanopore is one of the most widely used sensing orifices. Several review papers have already summarized the advances in the fabrication of solid-state nanopores and their applications on single-molecule sensing, DNA sequencing, genetics, medical diagnostics, and so on [93,94,95]. Generally speaking, a nanopore is typically of cylindrical [55,96,97,98] or conical [99,100,101,102] shape for use in RPS. In practice, most of the nanopore is of conical shape due to fabrication limitations. The conical nanopore is much more advantageous in sensitivity and accuracy due to the concentrated electric field at the entrance of a conical nanopore [99]. According to the material of a nanopore, it can be generally divided into two categories: biological nanopores and solid-state nanopores.

#### 5.1.1. Biological Nanopores

α-Hemolysin (α-HL) is one typical biological nanopore used for RPS [103]. The hydrophilic α-HL nanopore can self-assemble into a planar lipid membrane [104]. In 2012, Gopfrich et al. [105] fabricated an α-HL nanopore which is embedded in lipid bilayers. The fabrication procedures can be found elsewhere [106]. Firstly, nanocapillaries of 200 nm diameter were fabricated by using a laser pipet puller. Then, this nanocapillary was put on the baseplate and a charge of Giant Unilamellar Vesicles (GUVs) was introduced. A few seconds later, a vesicle cracked on the nanotip area to create a nanobilayer. The formation of the nanobilayer was detected by measuring the current drop. This was a novel technique for nanobilayer manufacturing. Compared with other conventional bilayer methods, it provided a fast and stable way to make a bilayer. 

However, the diameter of α-HL nanopores is only 1.4 nm, which is not suitable for the detection of some large molecules. To deal with this problem, some other biological membrane, such gareolysin [107], FhuA [108,109,110], and ClyA [111,112] hetero-oligomeric channels formed by NfpA and NfpB [113], were used. For biological nanopores, these are preferable for DNA sensing and sequencing. Although these nanopores achieved some success in a variety of applications, they had several weaknesses that hindered the advance of biological sensing in nanopores, for example, the low mechanical rigidity of the lipid membrane and shorter lifetime, etc. [114].

#### 5.1.2. Solid-State Nanopores

Compared with the biological nanopores, a solid-state nanopore is durable and can be easily fabricated into the desired geometries. Such merits are advantageous for commercial application [115].

##### (1) Glass

Nanopores made of glass have a high mechanical rigidity and chemical resistance and have been widely used for RPS [114,115,116,117,118,119,120]. The most widely used method for glass nanopore fabrication is by the pulling-and-etching method, which is simple and readily available with a relatively high resolution [117,120]. Surface modification techniques can also be applied to the nanopore to achieve some specific purposes. Such a surface charge-modulated RPS sensor was proven to be highly sensitive and selective for target particle detection [118].

He et al. [119] reported a novel surface modification method that can be reliably applied for selectively detecting uric acid (UA). To modify the nanopore, firstly the inner surface of the capillary tip was coated with a poly (l-histidine) (PLH) monolayer, which worked as the substrate for Au assembly. This was followed by in situ chemical reduction of AuCl_4_^−^. An Au nanofilm and 2-thiouracil (2-TU) were generated and coated on the inner surface of the glass nanopore. The binding of the target molecules with the functioned 2-TU will change the ionic current of the RPS system which can be used for quantitative detection of UA.

##### (2) Polymer Membranes

Polymer membranes were a good material for fabricating the nano-orifice due to their excellent optical properties [121,122]. The track-etched method was a popular method for fabricating a nano-orifice in polymer membranes [52,123] because it is cost-saving, reproducible, and provides easy precision controlling [123]. For example, the authors [52] developed a novel method to accurately control the geometry of the poly(ethylene terephthalate) (PET) nanopore by controlling the proportion of methanol in the etching solution. Their results showed that the pore diameters can be accurately controlled by applying different amount of methanol in the alkali solution. The base diameters of the PET membrane were 512 ± 30 nm and 928 ± 33 nm, with volume proportions of methanol of 0% and 10%, respectively. The maximum deviation of the base diameters was only 5.8%.

##### (3) Silicon Dioxide and Silicon Nitride

The silicon-based nanopore is another popular nanopore which can be fabricated by using the electron beam writing method [124,125,126]. The diameter of such a nanopore can be smaller than 10 nm [125]. A chemically etched SiN*_x_* nanopore was recently reported by Kwok [127,128]. The SiN*_x_* membrane was immersed in 3.6 M LiCl buffer solution at pH = 10, where the dielectric breakdown was controlled. The nanopores manufactured by this method showed a very high resolution (2.0 ± 0.5 nm in diameter) and over 95% reproducibility.

Yanagi et al. [129] fabricated a silicon nitride nanopore with a diameter of 1–2 nm, using the technique of multilevel pulse-voltage injection (MPVI). There is a trans chamber and a cis chamber across the 10 nm-thick Si_3_N_4_ membrane. Two Ag/AgCl electrodes were inserted into the two chambers and connected to an AC power source and an ammeter. The voltages were used to drill a nanopore across the membrane. The nanopore was generated when the measured current in this system exceeds the preset value.

##### (4) Graphene

Recently, advancements on fabricating graphene nanopores were reported [130,131,132]. Using electron beam writing, graphene nanopores with diameters less than 5 nm were achieved [131,133]. Besides the traditional electron beam writing method, Deng et al. [134] proposed a technique of fabricating a graphene nanopore of 5 nm in diameter with a helium ion microscope (HIM). For this method, there is a trimer to emit the helium ion beam under a high voltage at the top of an emitter. Under the action of the He^+^ ion, a graphene nanopore can be obtained. In that paper, they demonstrated a precise control of nanopore size and shape during the manufacturing process by controlling the dwell time of exposures.

### 5.2. PDMS Nanochannels

Nanochannels can be fabricated both in polymer and glass substrates. The two major advantages of using these nanochannels are being an integral part in a complex fluidic structure and allowing optical access to objects transported inside the channels. Fabricating a PDMS nanochannel is more attractive due to the distinctive advantage of PDMS in developing microfluidic and nanofluidic devices. Generally, a mold or master is needed for PDMS replica. Such a mold is normally fabricated by methods of the electron beam lithography (EBL) [135,136,137,138,139,140,141] and focused ion beam (FIB) [142,143,144,145,146,147,148,149]. For these two methods, the wavelength of the e-beam has a vital influence on the resolution of the nanochannel. However, the equipment is very expensive and not every lab can afford it [150].

**Figure 6:**
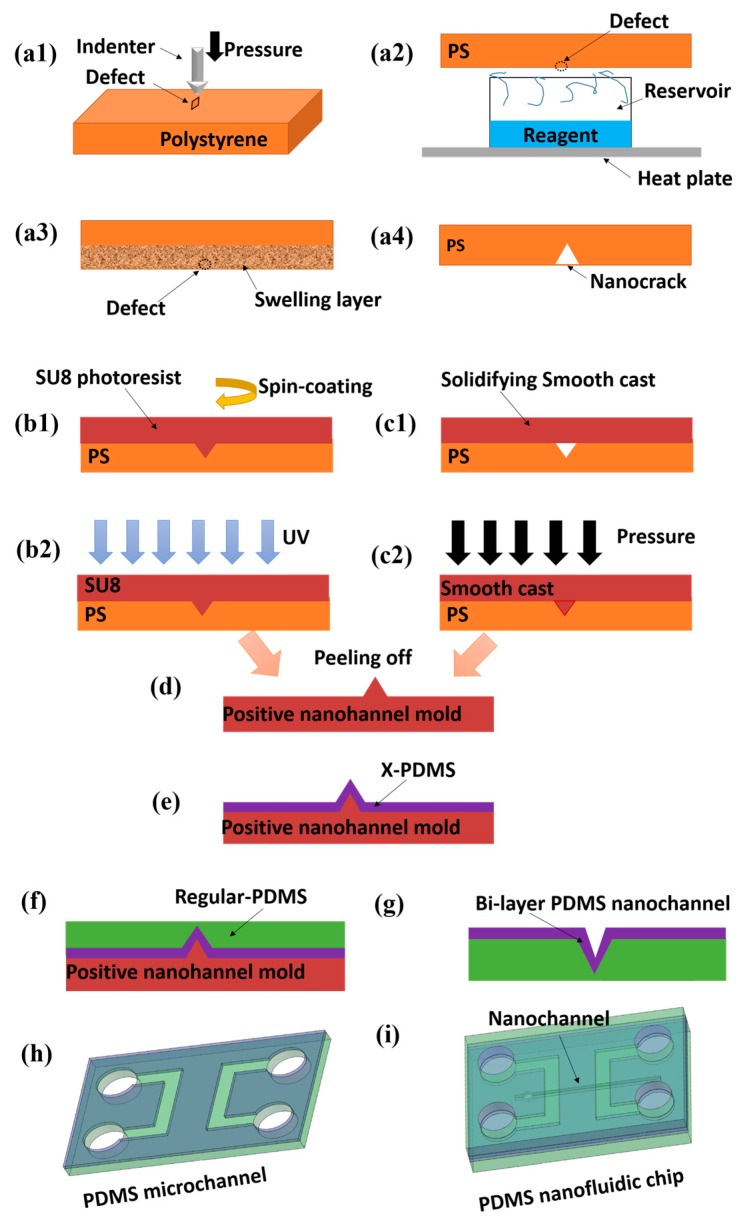
Procedures for polydimethylsiloxane (PDMS) nanochannel fabrication by using a solvent-induced nanocrack. (**a1**) making microdefects on a polystyrene slab by using an indenter of a micro-hardness testing system; (**a2**) absorption of the solvent; (**a3**) swelling of the polystyrene surface and initialization of nanocracks; (**a4**) nanocracks on the polystyrene surface. (**b1**) spin-coating of SU8 photoresist on the polystyrene slab with nanocracks; (**b2**) exposure the SU-8 layer to UV light. (**c1**) fabricating of solidifying smooth cast slab; (**c2**) nanoimprint by using a pressure gauge. (**d**)–(**i**) Show how to make a PDMS micro–nanofluidic chip by using the nanochannel mold: (**d**) nanochannel mold after peeling off process; (**e**) coating of x-PDMS on the nanochannel mold; (**f**) casting another layer of regular PDMS on the x-PDMS; (**g**) bi-layer PDMS nanochannel; (**h**) fabrication of bi-layer microchannel system; (**i**) PDMS micro–nanofluidic chip after bonding.

To develop a simpler and reliable PDMS nanochannel fabrication method, Peng and Li [151] reported fabricating a single nanocrack on a polymer substrate by using a solvent-induced technique (Figure 6). The minimal dimension of the nanocracks could reach nearly 64 nm in width and 17.4 nm in depth. Using this method, a PDMS nanochannel with a width of about 100 nm was fabricated [152]. The key procedure of this fabrication technique is the making of nanocracks firstly. The next step is to employ the soft lithography technique (SU8 photoresist) to copy this nanochannel mold. At last, the smooth cast slab is solidified and nanoimprinted by pressure gauge.

## 6. Outlook 

The latest development of microfuidic and nanofluidic RPS technologies within the last 6 years is reviewed in this paper. Some new and important phenomena both in microscale and nanoscale have been discovered, which greatly enriches the power of the Coulter principle. Researches on micro- and nano-RPS, both from the aspects of theory and application, still have many challenges and great potential. For example, one big question is the mathematic relationship between the resistance change and the sizes of a particle and a sensing channel at nanoscale, especially when the EDLs of the sensing channel are overlapped. Another challenge which prevents the wide application of the nano-RPS system is the integration of a microchannel with the nanochannel. While there are variety of nanochannel fabrication methods, as reviewed above, difficulties still exist for the integration of a nanochannel with a microchannel. Simple and reliable integration technology can greatly benefit both fundamental and applied research.

## Figures and Tables

**Table 1:** Relative resistance changes caused by particles passing a sensing orifice. *D* and *L* are the diameter and length of the sensing orifice, *d* is the diameter of the particle.

Relative Particle Diameter (*d/D*)	Relative Resistance Change (Δ*R/R*)
Infinite smaller diameter [38]	$\frac{d^{3}}{D^{2}L}$
Smaller diameter [39]	$\frac{d^{3}}{D^{2}L}\left[\frac{D^{2}}{2L^{2}}+\frac{1}{\sqrt{1+{\left(D/L\right)}^{2}}}\right]F\left(\frac{d^{3}}{D^{3}}\right)$
Medium diameter [40,41]	$\frac{d^{3}}{D^{2}L}\cdot \frac{1}{1-0.8{\left(d/D\right)}^{3}}$
Larger diameter [42]	$\frac{D}{L}\left[\frac{\arcsin(d/D)}{\sqrt{1-{\left(d/D\right)}^{2}}}-\frac{d}{D}\right]$

*Note:* $F(x)=1+1.264x+1.347x^{2}+0.648x^{3}+4.167x^{4}$ [43].
